# SNP variants associated with non-Hodgkin lymphoma (NHL) correlate with human leukocyte antigen (HLA) class II expression

**DOI:** 10.1038/srep41400

**Published:** 2017-01-31

**Authors:** Lik-Chin Ten, Yoon-Ming Chin, Mei-Chee Tai, Edmund Fui-Min Chin, Yat-Yuen Lim, Sujatha Suthandiram, Kian-Meng Chang, Tee-Chuan Ong, Ping-Chong Bee, Zahurin Mohamed, Gin-Gin Gan, Ching-Ching Ng

**Affiliations:** 1 Institute of Biological Sciences, Faculty of Science, University of Malaya, Kuala Lumpur, Malaysia; 2 Department of Medicine, Faculty of Medicine, University of Malaya, Kuala Lumpur, Malaysia; 3 Department of Pharmacology, Faculty of Medicine, University of Malaya, Kuala Lumpur, Malaysia; 4 Hematology Unit, Ampang Hospital, Kuala Lumpur, Malaysia

## Abstract

Large consortia efforts and genome-wide association studies (GWASs) have linked a number of genetic variants within the 6p21 chromosomal region to non-Hodgkin lymphoma (NHL). Complementing these efforts, we genotyped previously reported SNPs in the human leukocyte antigen (HLA) class I (rs6457327) and class II (rs9271100, rs2647012 and rs10484561) regions in a total of 1,145 subjects (567 NHL cases and 578 healthy controls) from two major ethnic groups in Malaysia, the Malays and the Chinese. We identified a NHL-associated (*P*_NHL_add_ = 0.0008; OR_NHL_add_ = 0.54; 95% CI = 0.37–0.77) and B-cell associated (*P*_Bcell_add_ = 0.0007; OR_Bcell_add_ = 0.51; 95% CI = 0.35–0.76) SNP rs2647012 in the Malaysian Malays. *In silico cis*-eQTL analysis of rs2647012 suggests potential regulatory function of nearby HLA class II molecules. Minor allele rs2647012-T is linked to higher expression of *HLA-DQB1,* rendering a protective effect to NHL risk. Our findings suggest that the HLA class II region plays an important role in NHL etiology.

Non-Hodgkin lymphomas (NHLs) are a heterogeneous group of neoplasms derived from B or T (natural killer) lymphocytes. It is the most common hematologic malignancy worldwide. About 90% of NHL cases are B-cell NHLs, with diffuse large B-cell lymphoma (DLBCL) and follicular lymphoma (FL) being the two most prevalent NHL subtypes in adults^1^. There are marked differences in the incidence patterns of NHL across geographical area or within the population itself, with higher prevalence in western countries in North America and Western Europe^2^. The overall incidence rate of NHL in Asia is much lower compare to that of Europe and North America^2^. In Malaysia, lymphoma ranks as the sixth most common cancer, with an overall incidence rate of 4.7 per 100,000 population^3^. Lymphoma is more prevalent among males (age-standardized rate per 100,000 people, ASR = 4.2) compared to females (ASR = 3.0). Malaysian Chinese were found to have higher incidence rate compared to Malays and Indians^3^.

The etiology of NHL remains largely unexplained, despite its dramatic worldwide rise in incidence in recent decades. Predisposition to autoimmune disorders^4^, immunodeficiency state of patients^5^, infectious agents such as human immunodeficiency virus (HIV)^6^, Epstein-Barr virus (EBV)^7^, *Helicobacter pylori*^8^ and human T-cell lymphotropic/leukemia virus-1 (HTLV-1) as well as exposure to chemical or pharmaceutical agents^9^,^10^ are few of the reported risk factors for NHL. Gene-environment interaction is also another potential risk factor of NHL^11^.

Single nucleotide polymorphisms (SNPs) are the most common polymorphisms in the human genome and are important markers for genetic studies. SNP alleles might alter gene expression levels and protein function, in the process, altering an individual’s susceptibility to disease including cancer^12^. In recent years, large consortia efforts and genome-wide association studies (GWASs) have identified a few susceptibility loci linked to NHL. The most compelling finding involves the discovery of SNPs in the human leukocyte antigen (HLA) region^13^,^14^,^15^. The HLA region maps to chromosome 6p21.3 in human. The primary function of HLA molecules is immune response. HLA class I molecules present endogenous antigens, and class II molecules present exogenous antigens to T cells, creating the “tri-molecular complex” (HLA-peptide-TCR) that initiates the immune response. The region contains more than 200 identified genes, over half of which are predicted to be expressed. Only some of the HLA region genes are involved in the immune response; in particular, the genes that encode the classical class I (A, B, and C) and class II (DR, DQ, and DP) antigens. Common genetic variants could alter the expression or function of HLA genes, disrupting the HLA molecules and subsequently its antigen presenting ability.

In this study, we genotyped four key SNPs in the HLA region to investigate their association with NHL in the Malaysian Malays and Chinese. rs6457327 is located near the *HLA-C* in the HLA class I region^14^; rs10484561 and rs2647012 are located in HLA class II region^13^, which are in high linkage disequilibrium with the extended haplotypes DRB1*01:01-DQA1*01:01-DQB1*05:01 and DRB1*15-DQA1*01-DQB1*06:02^16^; rs9271100 is located in HLA class II region and was associated with numerous autoimmune disease since its first discovery through the GWAS of systemic lupus erythematosus (SLE)^17^. Through this approach, we hope to identify a global NHL SNP, one that is linked to NHL in both Caucasian populations and the Malaysian population.

## Results

### Association between the SNPs and NHL susceptibility

Our NHL study comprised of 567 NHL patients and 578 healthy controls. Of these, 304 (53.6%) were Malay NHL patients and 263 (46.4%) were Chinese. The 578 healthy controls consist of 308 (53.3%) Malays and 270 (46.7%) Chinese. The average age of NHL patients were 54.6 years while controls were 44.2 years. Majority of NHL samples recruited from UMMC (n = 228) and Ampang Hospital (n = 339) were B-cell NHLs 496 (87.5%) with only 71 (12.5%) samples being T-cell/natural killer NHLs. Majority of B-cell NHLs were DLBCL (53.3%) and FL (14.6%) subtypes. Demographic characteristics of study subjects are listed in Table 1.

We evaluated the association of rs6457327, rs2647012 and rs10484561 with NHL in the Malaysian Malay and Chinese subjects. These SNPs were previously reported in NHL large consortia studies in Caucasian populations. We also included rs9271100, a prominent SNP located near *HLA-DRB1* constantly associated with autoimmune or autoimmune-like disease, a risk factor of NHL. In our statistical analysis, 3 genetic models, namely additive, dominant and recessive models, were evaluated. The different genetic models would enable us to identify the model that best represents correlation between SNP alleles and gene expression profiles.

We observed association for rs2647012 with all NHL types (*P*_NHL_add_ = 0.0008; OR_NHL_add_ = 0.54; 95% CI = 0.37–0.77) and B-cell NHL (*P*_Bcell_add_ = 0.0007; OR_Bcell_add_ = 0.51; 95% CI = 0.35–0.76) in the Malay samples (Table 2, Supplementary Table S1). SNP rs2647012 showed the strongest association in the additive genetic model. Association of all other SNPs with other ethnicities was not significant (*P* > *P*_Bonf_ 0.00083) (Supplementary Table S1). This association has marginal power >60% and shows no deviation from HWE (Table 2). All 4 SNPs in our study are independent markers due to very weak pairwise LD observed in both Malay and Chinese subjects (Supplementary Table S2).

There is a substantial age difference between NHL cases and controls in the Malay samples. We performed additional analysis to evaluate the confounding effects of age on the association of rs2647012. From our samples, we selected Malay NHL cases and controls in a younger age group (18–55 years) as well as in older subjects (removing controls age <40 years). For analysis of Malay subjects aged 18–55 years, average age between subjects were reduced for all NHL types [Case = 41.6 (±10.5); Ctrls = 39.4 (±8.9)] as well as B-cell NHL [Case = 42 (±10.4); Ctrls = 39.4 (±8.9)]. For analysis excluding controls aged <40 years, average age between subjects was also reduced for all NHL types [Case = 51.8 (±14.7); Ctrls = 48.3 (±7.7)] as well as B-cell NHL [Case = 52.6 (±14.5); Ctrls = 48.3 (±7.7)]. Details are listed in Table 3.

For all NHL types, Malay subjects from 18–55 years of age, the association of rs2647012 (*P*_NHL_add_ = 0.005408; OR_NHL_add_ = 0.55; 95% CI = 0.36–0.84) surpasses the nominal threshold of 0.05 but not the multiple testing threshold (*P*_Bonf_ = 0.00083). Likewise for B-cell NHL, rs2647012 (*P*_Bcell_add_ = 0.006009; OR_Bcell_add_ = 0.53; 95% CI = 0.34–0.83) is not significantly associated with NHL after multiple testing correction. The effect sizes remain consistent in direction with our initial unmatched cases and controls analysis (Table 2, Supplementary Table S3).

Similar results were observed for analysis excluding controls aged <40 years. In all NHL types for Malay subjects, the association of rs2647012 (*P*_NHL_add_ = 0.0045; OR_NHL_add_ = 0.56; 95% CI = 0.38–0.84) surpasses the nominal threshold of 0.05 but not after multiple testing correction. Likewise for B-cell NHL, rs2647012 (*P*_Bcell_add_ = 0.003657; OR_Bcell_add_ = 0.54; 95% CI = 0.36–0.82) is not significantly associated after multiple testing correction. The effect sizes remain consistent with our initial unmatched cases and controls. (Table 2, Supplementary Table S3).

**Figure 1:**
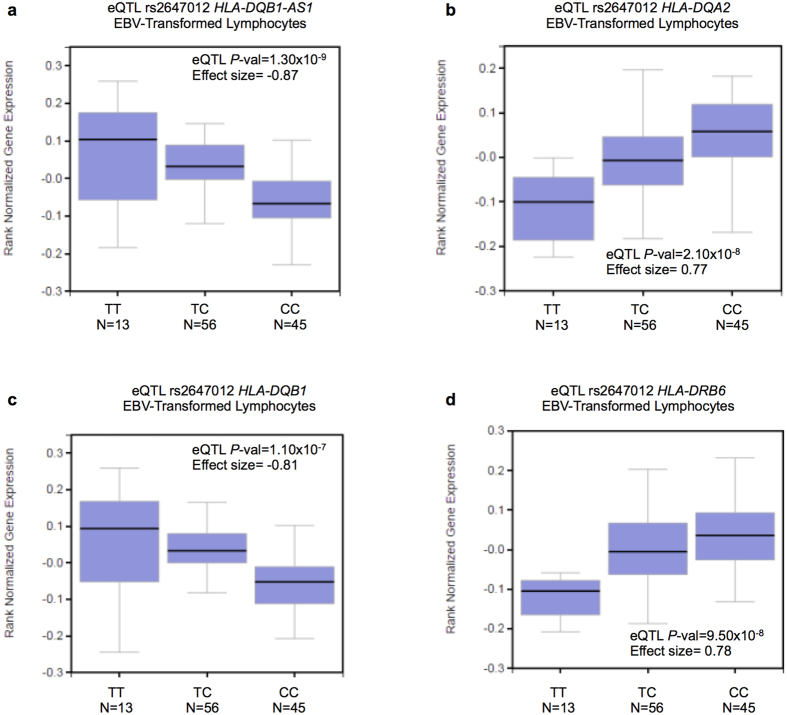
*cis*-eQTL analysis of NHL associated SNPs in EBV-transformed lymphocytes. Box-and-whisker plots showing correlation of rs2647012 C/T with expression levels of **(a)** *HLA-DQB1-AS1* **(b)** *HLA-DQA2* **(c)** *HLA-DQB1* **(d)** *HLA-DRB6*.

We evaluated potential regulatory function of rs2647012 through *in silico cis*-eQTL analysis. EBV-transformed lymphoblastoid cells were chosen as the closest model system to NHL and B-cell NHL. rs2647012 is correlated with *HLA-DQB1-AS1 (P*_FDR_*cis*-eQTL_ = 1.30 × 10^−9^; Effect size = −0.87), *HLA-DQA2 (P*_FDR_*cis*-eQTL_ = 2.10 × 10^−8^; Effect size = 0.77), *HLA-DQB1 (P*_FDR_*cis*-eQTL_ = 1.10 × 10^−7^; Effect size = −0.81) and *HLA-DRB6 (P*_FDR_*cis*-eQTL_ = 9.50 × 10^−8^; Effect size = 0.78) (Fig. 1a–d). Minor allele rs2647012-T is correlated with increased expression for *HLA-DQB1-AS1* and *HLA-DQB1* (Fig. 1a,c) and decreased expression for *HLA-DQA2* and *HLA-DRB6* (Fig. 1b,d). The correlation of rs2647012 with EBV cells expression profiles could be due to the EBV transformation process. Thus, we also evaluated *in silico cis*-eQTL of rs2647012 in other cell types. Our results show that rs2647012 genotypes were correlated with expression profiles of many other cell types, making it a global eQTL SNP (Supplementary Table S4).

## Discussion

We report the association of rs2647012, a HLA class II SNP with NHL and B-cell NHL in the Malaysian Malay population. The disparity in age between Malay NHL cases and controls poses a challenge to the reliability of our analysis. We have done additional analysis by looking at Malay NHL cases and controls in a younger age group (18–55 years) as well as in older subjects (removing controls age <40 years). From our results, we observed that the age difference between NHL cases and controls did not affect the association in our study. The effect size remained consistent across the sub-analysis and initial analysis. Matching subjects based on age would have decreased the power of our association. Hence, the need to use a larger sample size at the expense of disparate age groups.

Our study lacks genome-wide data to enable correction of population structure. Previous reports have suggested a binary clustering of the Malay population in Peninsular Malaysia, grouping Malaysian Malays based on northern and southern origins^18^. Thus, the association of rs2647012 could be confounded by presence of population structure in the Malaysian Malay population.

The effect size and direction of rs2647012 in the Malaysian Malays are consistent with previous reports in Caucasian populations^15^, rendering a protective effect (Supplementary Table S5). The minor allele frequency differs, as rs2647012-T is less prevalent in Malays (MAF = 0.18) compared to Caucasian populations (MAF = 0.48). Other SNPs also displayed a similar trend. The association of rs6773854 was not replicated in our preliminary study despite its strong association to B-cell NHL in the Chinese population^19^.

*In silico cis*-eQTL analysis of rs2647012 suggests potential regulatory function of nearby HLA class II molecules. rs2647012-T is linked to higher expression of *HLA-DQB1*, an observation also reported by Sille and colleagues^20^. rs2647012-T is linked with lower expression of *HLA-DQA2*, contradicting the protective effect rendered by rs2647012-T. Our results could be important as increased *HLA-DQB1* expression may enhance antigen presentation, immune surveillance and the elimination of tumor cells. Low levels of *HLA-DR/DQ* expression have been linked to decreased immune response in lymphoma cells^21^. HLA class II molecules are expressed in antigen presenting cells such as B-lymphocytes, and act to present exogenous antigens to CD4 + helper T-cells. Change in expression of these HLA class II molecules, modulated by SNPs, may compromise efficiency of antigen presentation.

## Conclusion

We report the association of rs2647012, a HLA class II SNP with NHL and B-cell NHL in the Malaysian Malay population. Through *in silico* eQTL analysis, rs2647012 genotypes were shown to correlate with expression levels of nearby HLA class II molecules. This report represents the largest sample size to date for NHL associations in a Malaysian population after our previous study on IL-10 promoter SNPs^22^. This report is also one of few blood cancer association reports in south Asian populations^19^,^23^. The HLA class II region is highly polymorphic with extended LD blocks. Therefore, future work would focus on fine mapping the HLA class II region as well as genotyping the HLA class II alleles to identify potential causal variants. We could also expand our analysis to include immune-related pathways of HLA class II molecules as well as the regulatory function of SNPs in modulating binding of transcription factors in promoter regions.

## Materials and Methods

### Study subjects and clinical characteristics

A total of 567 NHL patients (304 Malays and 263 Chinese) were recruited from University Malaya Medical Centre (UMMC) and Ampang Hospital from April 2007 to July 2014. NHL types and subtypes were classified according to the World Health Organization (WHO) 2008 classification system. The criteria for NHL case recruitment were adult patients above the age of 18 years old, HIV-negative NHL patients and patients without undergoing haematopoietic stem cell transplantation. All 578 unrelated healthy controls (308 Malays and 270 Chinese) were aged between 18–85 years old. Younger control subjects were recruited from various blood donation campaigns held in UMMC during the same period of time while older control subjects were collected from outpatient clinics in UMMC. All controls were free from any underlying malignancies. Both NHL patients and healthy controls were unrelated self-reported ethnicity of Malay or Chinese descent. Written informed consent was obtained from all patients and healthy controls prior to blood/ buccal samples collection. This study was approved by UMMC Ethics committee (Reference no.: 565.7) and also registered under National Medical Research Register (NMRR; Research ID: 21855) from Ministry of Health (MOH) Malaysia. All the methods were performed in accordance with the relevant guidelines and regulations from ethical committees of both centers.

### Genomic DNA extraction

Genomic DNA was extracted from peripheral blood/buccal cells using QIAamp DNA Blood Midi Kit or QIAamp DNA Blood Mini Kit for buccal swabs (QIAGEN Inc, Germantown, MD, USA) following manufacturer’s protocol. The quality and quantity of extracted DNA was determined using NanoDrop^®^ ND-1000 spectrophotometer (Thermo Fisher Scientific Inc., Wilmington, DE, USA).

### TaqMan SNP genotyping method

Genotyping was done using TaqMan^®^ SNP genotyping assay (Applied Biosystems, Foster City, CA) and performed on the QuantStudio™ 12 K Flex Real Time PCR System (Applied Biosystems, Foster City, CA). SNPs showing genotype call rates >95% were retained for statistical analysis. To evaluate concordance of the genotyping calls, 5% of the samples for each SNP were randomly selected for re-genotyping.

### Statistical analysis of genotyping data

Statistical analysis was performed separately for Malay and Chinese ethnic groups. Within each ethnic group, SNP association was calculated for all NHL types as well as NHL subtypes of B-cell NHL, T-cell NHL, DLBCL and FL. Logistic regression was performed using an additive model, adjusting for age and gender. Odds ratio (OR) and 95% confidence intervals (CI) were calculated referencing the minor allele. All SNPs were evaluated for deviation from the Hardy-Weinberg equilibrium (HWE) in control subjects. SNPs deviating from HWE (*P*_HWE_ < 0.05) were excluded from our analysis. We applied the Bonferroni method for multiple testing correction of the type 1 error. We corrected for 4 independent SNPs across 3 sample groups and 5 NHL types (α = 0.05/60 = 0.00083). SNPs showing *P* < 0.00083 were considered significantly associated. To address the disparity in age between NHL cases and controls, we performed additional analysis by selecting Malay NHL cases and controls in a younger age group (18–55 years) as well as in older subjects (removing controls age <40 years). Pairwise linkage disequilibrium (LD) between all 4 SNPs was calculated to assess linkage or independence of the 4 variants. All statistical analysis were performed in PLINK v1.07 unless stated otherwise (see URL)^24^. Meta-analysis was performed in META (see URL) assuming a random-effects model to estimate the combined OR and *P*-value of SNPs in the combined analysis of Malay and Chinese subjects. A random-effects model was chosen considering the different ancestry of the Malay and Chinese subjects. Power analysis of all SNPs was calculated using QUANTO v1.2.4 (see URL)^25^. SNPs with power associations <70% was considered underpowered. Linkage disequilibrium was calculated using both Pearson’s correlation (*r*^2^) and Lewontin’s D-prime (*D*′).

### *Cis*-eQTL analysis of NHL associated SNPs

Potential regulatory function of NHL SNPs was evaluated through *in silico e*QTL from GTEx version 6 (see URL)^26^. We focused on EBV-transformed lymphocytes, the most relevant cell type for our study. *e*QTL from other cell types were evaluated as well to ascertain the global regulatory potential of our SNPs. Details of the *cis*-eQTL analysis have been mentioned elsewhere^26^. Briefly, *cis*-eQTL was performed in Matrix eQTL^27^ using linear regression, assuming an additive model, adjusting for 3 principal components and PEER factors. The *cis*-eQTL was defined as a window of +/−1MB from the transcription start site (TSS). Storey FDR is used to correct for multiple hypothesis, with *cis*-eQTLs chosen as significant for q-values ≤ 0.05.

### URL

PLINK v1.07 http://pngu.mgh.harvard.edu/~purcell/plink/

META https://mathgen.stats.ox.ac.uk/genetics_software/meta/meta.html

QUANTO v1.2.4 http://biostats.usc.edu/Quanto.html

GTEx V6 http://www.gtexportal.org/home/

## Additional Information

**How to cite this article**: Ten, L.-C. *et al*. SNP variant associated with non-Hodgkin lymphoma (NHL) correlate with human leukocyte antigen (HLA) class II expression. *Sci. Rep.* **7**, 41400; doi: 10.1038/srep41400 (2017).

**Publisher's note:** Springer Nature remains neutral with regard to jurisdictional claims in published maps and institutional affiliations.

## Supplementary Material

Supplementary Tables

## Figures and Tables

**Table 1:** Demographics of study subjects.

	Malay (n = 612)		Chinese (n = 533)		Total (n = 1,145)	
	Case (%)	Control (%)	Case (%)	Control (%)	Case (%)	Control (%)
**Male**	166 (54.6%)	170 (55.2%)	147 (55.9%)	156 (57.8%)	313 (55.2%)	326 (56.4%)
**Female**	138 (45.4%)	138 (44.8%)	116 (44.1%)	114 (42.2%)	254 (44.8%)	252 (43.6%)
**Age**^a^	51.8 (±14.7)	39.7 (±10.0)	57.1 (±14.4)	49.1 (±12.1)	54.6 (±14.7)	44.2 (±14.1)
**Age range**	18–91	19–85	18–87	18–84	18–91	18–85
**B-cell NHL**	268 (88.2%)	—	228 (86.7%)	—	496 (87.5%)	—
DLBCL	173 (56.9%)	—	129 (49.0%)	—	302 (53.3%)	—
FL	38 (12.5%)	—	45 (17.1%)	—	83 (14.6%)	—
Others^b^	57 (18.8%)	—	54 (20.5%)	—	111 (19.6%)	—
**T-cell NHL**^c^	36 (11.8%)	—	35 (13.3%)	—	71 (12.5%)	—
**Total**	304 (100%)	308 (100%)	263 (100%)	270 (100%)	567 (100%)	578 (100%)

*Abbreviations: NHL, non-Hodgkin’s lymphoma; DLBCL, diffuse large B-cell lymphoma; FL, follicular lymphoma.

^a^Age at interviewed for cases and controls- Mean (Standard deviation).

^b^Others: Other types of B-cell NHL except DLBCL and FL.

^c^T-cell NHL: Included all the subtypes of T-cell NHL.

**Table 2:** Association of rs2647012 with NHL in Malay study subjects according to different age groups.

NHL Type	SNP+	Position	Nearby genes	Subjects	Sample size (Case/Ctrl)	MAF (Case/Ctrl)	OR (95% CI)	*P*-value*	Genetic model	Power
All NHL	rs2647012	Chr 6:	30 kb 5′ of	ALL	304/308	0.11/0.18	0.54 (0.37–0.77)	0.0008	Additive	64.1%
	C > T	32,696,681	*HLA-DQB1*	Case & control 18–55 y/o	170/195	0.11/0.19	0.55 (0.36–0.84)	0.005408	Additive	30.8%
	Smedby *et al*.^15^			Control > 40 y/o	304/139	0.11/0.18	0.56 (0.38–0.84)	0.0045	Additive	30.0%
B-cell NHL	rs2647012	Chr 6:	30 kb 5′ of	**ALL**	**268/308**	**0.11/0.18**	**0.51 (0.35–0.76)**	**0.0007**	Additive	**70.0%**
	C > T	32,696,681	*HLA-DQB1*	Case & control 18–55 y/o	145/195	0.11/0.19	0.53 (0.34–0.83)	0.006009	Additive	31.2%
	Smedby *et al*.^15^			Control > 40 years	268/139	0.11/0.18	0.54 (0.36–0.82)	0.003657	Additive	33.0%

^+^rs2647012 shows no deviation from HWE (*P*_NHL_HWE_ = 0.2375; *P*_Bcell_HWE_ = 0.2375).

^*^SNPs showing *P* < 0.00083 is considered significantly associated and highlighted in black. Bonferroni threshold includes correcting for 4 independent SNPs across 3 sample groups and 5 NHL types.

**Table 3:** Demographics of Malay study subjects according to different age groups.

Age group	Age range	NHL Type	Average age (SD)^a^	Median age	Case	Control
**Case & Control 18–55**	Case = 18–55;Ctrls = 19–55	All NHL	Case = 41.6 (±10.5);Ctrls = 39.4 (±8.9)	Case = 45;Ctrls = 39	Male = 86;Female = 84;Total = 170	Male = 100;Female = 95;Total = 195
		B-cell NHL	Case = 42 (±10.4);Ctrls = 39.4 (±8.9)	Case = 45;Ctrls = 39	Male = 75;Female = 70;Total = 145	Male = 100;Female = 95;Total = 195
**Control > 40**	Case = 18–91;Ctrls = 40–95	All NHL	Case = 51.8 (±14.7);Ctrls = 48.3 (±7.7)	Case = 54;Ctrls = 47	Male = 166;Female = 138;Total = 304	Male = 86;Female = 53;Total = 139
		B-cell NHL	Case = 52.6 (±14.5);Ctrls = 48.3 (±7.7)	Case = 54.5;Ctrls = 47	Male = 147;Female = 121;Total = 268	Male = 86;Female = 53;Total = 139

*Abbreviations: NHL, non-Hodgkin’s lymphoma; Ctrls, controls.

^a^Age at interviewed for cases and controls- Mean (Standard deviation).
