# Supplementary material for: SNP variants associated with non-Hodgkin lymphoma (NHL) correlate with human leukocyte antigen (HLA) class II expression
Source: Sci Rep. 2017 Jan 31;7:41400. doi: 10.1038/srep41400 (PMC5282517; doi:10.1038/srep41400)
Supplement: Supplementary Tables [file srep41400-s1.pdf]

Title: SNP variants associated with non-Hodgkin lymphoma (NHL) correlate with human leukocyte antigen (HLA) class II expression

Lik-Chin Ten[1❖], Yoon-Ming Chin[1❖], Mei-Chee Tai[1], Edmund Fui-Min Chin[2], Yat-Yuen Lim[1], Sujatha Suthandiram[3], Kian-Meng Chang[4], Tee-Chuan Ong[4], Ping-Chong Bee[2], Zahurin Mohamed[3], Gin-Gin Gan[2*] & Ching-Ching Ng[1*]

[1]Institute of Biological Sciences, Faculty of Science, University of Malaya, Kuala Lumpur, Malaysia.
[2]Department of Medicine, Faculty of Medicine, University of Malaya, Kuala Lumpur, Malaysia.
[3]Department of Pharmacology, Faculty of Medicine, University of Malaya, Kuala Lumpur, Malaysia.
[4]Hematology Unit, Ampang Hospital, Kuala Lumpur, Malaysia.

❖These authors contributed equally.
*Shared last author.
Correspondence:
Ching-Ching Ng, Institute of Biological Sciences, Faculty of Science, University of Malaya, 50603 Kuala Lumpur, Malaysia; Tel: +60379675872; Fax: +60379675908; E-mail: ccng@um.edu.my
Gin-Gin Gan, Department of Medicine, Faculty of Medicine, University of Malaya, 50603 Kuala Lumpur,
Malaysia; Tel: +60379492429; Fax: +60379556936; E-mail: gangg@ummc.edu.my

Supplementary Table S1. SNPs associated with different NHL subtypes in Malaysian subjects assuming different genetic models

NHL Type	SNP	CHR	GRCh38.p2	Subjects	Case MAF	Control MAF	OR_{add} (95% CI)	P_{add}	OR_{dom} (95% CI)	P_{dom}	OR_{rec} (95% CI)	P_{rec}	P_{HWE}	Reference
All NHL	rs6457327	6	31106253	Malay	0.39	0.41	0.95 (0.74-1.22)	0.6612	0.95 (0.66-1.37)	0.7864	0.89 (0.56-1.43)	0.6347	0.5572	Skibola et al., 2009; Conde et al., 2010
Malay NHL=304, Malay control=308	C>A			Chinese	0.33	0.35	0.86 (0.66-1.12)	0.2679	0.75 (0.52-1.07)	0.1111	1.04 (0.6-1.79)	0.9002	1	
Chinese NHL=263, Chinese control=270				Combined	0.36	0.38	0.9 (0.75-1.09)	0.279879	0.84 (0.65-1.09)	0.184558	0.95 (0.67-1.36)	0.781511	-	
Combined NHL=567; Combined control=578	rs9271100	6	32608701	Malay	0.34	0.32	1.07 (0.83-1.39)	0.5945	1.11 (0.78-1.59)	0.5564	1.06 (0.61-1.83)	0.8318	0.3545	Han et al., 2009; Liu et al., 2015
	C>T			Chinese	0.20	0.17	1.28 (0.92-1.78)	0.1433	1.26 (0.86-1.83)	0.2384	2.09 (0.72-6.08)	0.1754	0.6622	
				Combined	0.28	0.25	1.15 (0.94-1.41)	0.185667	1.18 (0.91-1.53)	0.215955	1.27 (0.71-2.28)	0.426746	-	
	rs2647012	6	32696681	Malay	0.11	0.18	**0.54 (0.37-0.77)**	**0.0007959**	0.52 (0.34-0.79)	0.001991	0.26 (0.08-0.87)	0.02896	0.2375	Smedby et al., 2011
	C>T			Chinese	0.21	0.22	0.96 (0.7-1.31)	0.7851	1 (0.69-1.44)	0.9829	0.73 (0.3-1.74)	0.4759	0.8562	
				Combined	0.16	0.20	0.72 (0.41-1.27)	0.262208	0.72 (0.38-1.37)	0.322659	0.48 (0.18-1.28)	0.140112	-	
	rs10484561	6	32697643	Malay	0.19	0.14	1.31 (0.94-1.82)	0.1118	1.33 (0.9-1.96)	0.1581	1.79 (0.68-4.73)	0.2377	0.4655	Conde et al., 2010
	T>G			Chinese	0.03	0.03	1.14 (0.56-2.31)	0.7179	1.14 (0.56-2.31)	0.7179	NA	NA	1	
				Combined	0.12	0.09	1.28 (0.95-1.72)	0.110695	1.28 (0.91-1.8)	0.159102	1.79 (0.68-4.73)	0.237721	-	
B-cell NHL	rs6457327	6	31106253	Malay	0.3825	0.4091	0.93 (0.72-1.21)	0.597	0.93 (0.63-1.36)	0.6967	0.88 (0.54-1.45)	0.6214	0.5572	Skibola et al., 2009; Conde et al., 2010
Malay B-cell=268, Malay control=308	C>A			Chinese	0.3158	0.35	0.82 (0.62-1.08)	0.159	0.7 (0.48-1.02)	0.05976	0.98 (0.55-1.75)	0.9575	1	
Chinese B-cell=228, Chinese control=270				Combined	0.3518	0.3815	0.88 (0.72-1.06)	0.17667	0.8 (0.61-1.06)	0.121026	0.92 (0.63-1.35)	0.682635	-	
Combined B-cell=496; Combined control=578	rs9271100	6	32608701	Malay	0.3333	0.3179	0.99 (0.76-1.31)	0.9651	1 (0.69-1.46)	0.9864	0.97 (0.54-1.72)	0.9055	0.3545	Han et al., 2009; Liu et al., 2015
	C>T			Chinese	0.193	0.166	1.22 (0.86-1.72)	0.2636	1.16 (0.78-1.72)	0.4638	2.3 (0.76-6.95)	0.1385	0.6622	
				Combined	0.2687	0.2465	1.08 (0.87-1.33)	0.508344	1.07 (0.82-1.41)	0.607226	1.31 (0.58-2.94)	0.518719	-	
	rs2647012	6	32696681	Malay	0.1063	0.1826	**0.51 (0.35-0.76)**	**0.0007103**	0.5 (0.32-0.78)	0.002302	0.2 (0.05-0.76)	0.01784	0.2375	Smedby et al., 2011
	C>T			Chinese	0.1974	0.2184	0.84 (0.6-1.16)	0.2838	0.81 (0.55-1.2)	0.2918	0.78 (0.32-1.94)	0.5943	0.8562	
				Combined	0.1482	0.1995	0.66 (0.41-1.07)	0.0921189	0.65 (0.41-1.03)	0.0673257	0.43 (0.11-1.63)	0.215616	-	
	rs10484561	6	32697643	Malay	0.1866	0.1352	1.33 (0.94-1.87)	0.1048	1.33 (0.88-2.01)	0.1732	2.02 (0.75-5.43)	0.165	0.4655	Conde et al., 2010
	T>G			Chinese	0.03509	0.03358	1.24 (0.59-2.58)	0.5733	1.24 (0.59-2.58)	0.5733	NA	NA	1	
				Combined	0.1169	0.08783	1.31 (0.96-1.79)	0.0876583	1.31 (0.91-1.87)	0.143328	2.02 (0.75-5.43)	0.1649	-	
DLBCL	rs6457327	6	31106253	Malay	0.3757	0.4091	0.9 (0.67-1.2)	0.4663	0.85 (0.55-1.3)	0.4453	0.9 (0.51-1.56)	0.698	0.5572	Skibola et al., 2009; Conde et al., 2010
Malay DLBCL=173, Malay control=308	C>A			Chinese	0.3062	0.35	0.79 (0.57-1.1)	0.1607	0.71 (0.46-1.1)	0.1251	0.82 (0.41-1.65)	0.5793	1	
Chinese DLBCL=129, Chinese control=270				Combined	0.346	0.3815	0.85 (0.68-1.06)	0.139726	0.78 (0.57-1.05)	0.105985	0.87 (0.56-1.34)	0.516442	-	
Combined DLBCL=302; Combined control=578	rs9271100	6	32608701	Malay	0.3314	0.3179	0.97 (0.71-1.31)	0.8234	0.94 (0.62-1.42)	0.7628	1 (0.53-1.87)	0.994	0.3545	Han et al., 2009; Liu et al., 2015
	C>T			Chinese	0.186	0.166	1.17 (0.78-1.75)	0.4512	1.12 (0.71-1.77)	0.6354	2.03 (0.57-7.23)	0.2734	0.6622	
				Combined	0.2691	0.2465	1.03 (0.81-1.32)	0.786371	1.01 (0.75-1.38)	0.926018	1.15 (0.65-2.02)	0.631035	-	
	rs2647012	6	32696681	Malay	0.1069	0.1826	0.53 (0.35-0.82)	0.003938	0.51 (0.31-0.83)	0.007224	0.27 (0.07-1.1)	0.06766	0.2375	Smedby et al., 2011
	C>T			Chinese	0.2171	0.2184	0.94 (0.65-1.37)	0.7479	0.93 (0.59-1.45)	0.7425	0.93 (0.34-2.58)	0.8948	0.8562	
				Combined	0.154	0.1995	0.72 (0.41-1.25)	0.237925	0.69 (0.39-1.25)	0.222969	0.55 (0.17-1.85)	0.336137	-	
	rs10484561	6	32697643	Malay	0.1734	0.1352	1.22 (0.84-1.78)	0.2995	1.15 (0.72-1.83)	0.5531	2.26 (0.78-6.54)	0.1317	0.4655	Conde et al., 2010
	T>G			Chinese	0.03876	0.03358	1.29 (0.56-2.97)	0.5443	1.29 (0.56-2.97)	0.5443	NA	NA	1	
				Combined	0.1159	0.08783	1.23 (0.87-1.74)	0.232713	1.18 (0.79-1.77)	0.416735	2.26 (0.78-6.54)	0.131783	-	
FL	rs6457327	6	31106253	Malay	0.3947	0.4091	1.01 (0.6-1.72)	0.9636	0.75 (0.34-1.66)	0.4808	1.56 (0.62-3.92)	0.3462	0.5572	Skibola et al., 2009; Conde et al., 2010
Malay T-cell=36, Malay control=308	C>A			Chinese	0.3222	0.35	0.81 (0.49-1.34)	0.4116	0.66 (0.33-1.3)	0.2292	1.05 (0.39-2.84)	0.9191	1	
Chinese FL=45, Chinese control=270				Combined	0.3554	0.3815	0.9 (0.62-1.3)	0.574112	0.7 (0.41-1.17)	0.170429	1.3 (0.66-2.56)	0.447804	-	
Combined FL=83; Combined control=578	rs9271100	6	32608701	Malay	0.3158	0.3179	0.98 (0.56-1.75)	0.9579	1.32 (0.6-2.89)	0.4935	0.42 (0.1-1.79)	0.2398	0.3545	Han et al., 2009; Liu et al., 2015
	C>T			Chinese	0.2222	0.166	1.47 (0.79-2.73)	0.2212	1.34 (0.66-2.73)	0.4213	4.29 (0.76-24.33)	0.1004	0.6622	
				Combined	0.2651	0.2465	1.18 (0.78-1.8)	0.428752	1.33 (0.78-2.25)	0.29077	1.27 (0.13-12.41)	0.83519	-	
	rs2647012	6	32696681	Malay	0.1184	0.1826	0.44 (0.19-1.05)	0.06298	0.48 (0.19-1.22)	0.1222	NA	NA	0.2375	Smedby et al., 2011
	C>T			Chinese	0.1556	0.2184	0.64 (0.33-1.24)	0.1878	0.6 (0.28-1.25)	0.1721	0.63 (0.08-5.13)	0.6632	0.8562	
				Combined	0.1386	0.1995	0.56 (0.33-0.94)	0.0292772	0.55 (0.31-0.98)	0.0424643	0.63 (0.08-5.13)	0.663202	-	
	rs10484561	6	32697643	Malay	0.2632	0.1352	2.7 (1.38-5.27)	0.003641	3.5 (1.54-7.97)	0.002886	2.82 (0.44-17.94)	0.2731	0.4655	Conde et al., 2010
	T>G			Chinese	0.03333	0.03358	1.46 (0.38-5.52)	0.5817	1.46 (0.38-5.52)	0.5817	NA	NA	1	
				Combined	0.1386	0.08783	2.38 (1.31-4.34)	0.00441769	2.66 (1.2-5.9)	0.0164136	2.82 (0.44-17.94)	0.273113	-	
T-cell NHL	rs6457327	6	31106253	Malay	0.4167	0.4091	1.04 (0.63-1.72)	0.8709	1.14 (0.54-2.42)	0.7345	0.94 (0.37-2.42)	0.9012	0.5572	Skibola et al., 2009; Conde et al., 2010
Malay T-cell=36, Malay control=308	C>A			Chinese	0.3857	0.35	1.11 (0.66-1.86)	0.7058	1.16 (0.56-2.43)	0.6871	1.1 (0.39-3.08)	0.8631	1	
Chinese T-cell=35, Chinese control=270				Combined	0.4014	0.3815	1.07 (0.75-1.54)	0.705415	1.15 (0.68-1.95)	0.599963	1.01 (0.5-2.02)	0.980781	-	
Combined T-cell=71; Combined control=578	rs9271100	6	32608701	Malay	0.4306	0.3179	1.58 (0.96-2.6)	0.06951	2.18 (1.02-4.68)	0.04521	1.45 (0.55-3.85)	0.4546	0.3545	Han et al., 2009; Liu et al., 2015
	C>T			Chinese	0.25	0.166	1.86 (0.98-3.51)	0.0565	2.11 (1.01-4.41)	0.0464	1.57 (0.17-14.13)	0.6881	0.6622	
				Combined	0.3429	0.2465	1.68 (1.14-2.49)	0.00921836	2.15 (1.26-3.65)	0.0047368	1.47 (0.6-3.58)	0.396986	-	
	rs2647012	6	32696681	Malay	0.125	0.1826	0.61 (0.3-1.27)	0.1844	0.55 (0.24-1.29)	0.1716	0.55 (0.07-4.5)	0.5767	0.2375	Smedby et al., 2011
	C>T			Chinese	0.3143	0.2184	1.7 (0.97-2.98)	0.06592	2.54 (1.22-5.29)	0.01288	0.5 (0.06-4.04)	0.5174	0.8562	
				Combined	0.2183	0.1995	1.05 (0.39-2.84)	0.929835	1.2 (0.27-5.35)	0.806869	0.53 (0.12-2.31)	0.393728	-	
	rs10484561	6	32697643	Malay	0.1944	0.1352	1.48 (0.78-2.8)	0.2309	1.68 (0.8-3.54)	0.1741	1.05 (0.12-9.5)	0.9667	0.4655	Conde et al., 2010
	T>G			Chinese	0.02857	0.03358	0.86 (0.19-3.94)	0.8439	0.86 (0.19-3.94)	0.8439	NA	NA	1	
				Combined	0.1127	0.08783	1.36 (0.76-2.45)	0.303964	1.47 (0.75-2.88)	0.256848	1.05 (0.12-9.51)	0.966758	-	

SNPs showing *P*<0.00083 is considered significantly associated and highlighted in black. Bonferroni thresold includes correcting for 4 independent SNPs across 3 sample groups and 5 NHL types.
Best SNPs *P*-value that do not overcome the Bonferonni threshold are highlighted in grey

Supplementary Table S2. Linkage disequilibrium of 4 SNPs in all NHL types for Chinese and Malay cohorts.

Chinese All NHL type Linkage Disequilibrium

	rs6457327	rs9271100	rs2647012	rs10484561
rs6457327		0.0295923	0.00116093	0.00978653
rs9271100	0.25955		0.0117324	0.0297918
rs2647012	0.0905969	0.433795		0.00696627
rs10484561	0.740596	0.442177	0.847419	

Malay All NHL type Linkage Disequilibrium

	rs6457327	rs9271100	rs2647012	rs10484561
rs6457327		0.0032436	0.0268834	0.0027598
rs9271100	0.0655956		0.0521176	0.218162
rs2647012	0.49006	0.791671		0.0263703
rs10484561	0.147213	0.747961	0.90249	

Pearson's correlation, r^2

Lewontin's D-prime, D'

Supplementary Table S3. SNPs associated with different NHL subtypes in Malaysian subjects with control samples aged more than 40 years.

NHL Type	SNP	CHR	GRCh38.p2	Subjects	Age groups	Case MAF	Control MAF	OR_{add} (95% CI)	P_{add}	OR_{dom} (95% CI)	P_{dom}	OR_{rec} (95% CI)	P_{rec}	Reference
All NHL	rs2647012 C>T	6	32696681	Malay	ALL	0.11	0.18	**0.54 (0.37-0.77)**	**0.0007959**	0.52 (0.34-0.79)	0.001991	0.26 (0.08-0.87)	0.02896	Smedby et al., 2011
					Control >40	0.11	0.18	**0.56 (0.38-0.84)**	**0.0045**	0.54 (0.34-0.87)	0.01053	0.29 (0.09-0.97)	0.04353	
					Case & control 18-55	0.11	0.19	**0.55 (0.36-0.84)**	**0.005408**	0.54 (0.34-0.88)	0.01322	0.22 (0.05-1.01)	0.05101	
B-cell NHL	rs2647012 C>T	6	32696681	Malay	ALL	0.11	0.18	**0.51 (0.35-0.76)**	**0.0007103**	0.5 (0.32-0.78)	0.002302	0.2 (0.05-0.76)	0.01784	Smedby et al., 2011
					Control >40	0.11	0.18	**0.54 (0.36-0.82)**	**0.003657**	0.53 (0.33-0.86)	0.009701	0.25 (0.07-0.9)	0.03347	
					Case & control 18-55	0.11	0.19	**0.53 (0.34-0.83)**	**0.006009**	0.54 (0.33-0.9)	0.01858	0.12 (0.02-0.98)	0.04775	

SNPs showing $P<0.00083$ is considered significantly associated and highlighted in black. Bonferroni thresold includes correcting for 4 independent SNPs across 3 sample groups and 5 NHL types.
Best SNPs *P*-value that do not overcome the Bonferroni threshold are highlighted in grey

Supplementary Table S4. eQTL of NHL-associated rs2647012 in whole blood and EBV-transformed lymphocytes from GTEx

Gencode Id	Gene Symbol	SNP Id	Synonym SNP	Allele	Ref/Alt allele	*P*-Value	Effect Size	Tissue
ENSG00000223534.1	*HLA-DQB1-AS1*	rs2647012	rs116393447	C>T	T/C	1.30E-09	-0.87	Cells - EBV-transformed lymphocytes
ENSG00000237541.3	*HLA-DQA2*	rs2647012	rs116393447	C>T	T/C	2.10E-08	0.77	Cells - EBV-transformed lymphocytes
ENSG00000229391.3	*HLA-DRB6*	rs2647012	rs116393447	C>T	T/C	9.50E-08	0.78	Cells - EBV-transformed lymphocytes
ENSG00000179344.12	*HLA-DQB1*	rs2647012	rs116393447	C>T	T/C	1.10E-07	-0.81	Cells - EBV-transformed lymphocytes
ENSG00000179344.12	*HLA-DQB1*	rs2647012	rs116393447	C>T	T/C	1.30E-34	-0.93	Adipose - Subcutaneous
ENSG00000229391.3	*HLA-DRB6*	rs2647012	rs116393447	C>T	T/C	2.90E-27	0.88	Adipose - Subcutaneous
ENSG00000223534.1	*HLA-DQB1-AS1*	rs2647012	rs116393447	C>T	T/C	5.40E-25	-0.81	Adipose - Subcutaneous
ENSG00000237541.3	*HLA-DQA2*	rs2647012	rs116393447	C>T	T/C	6.20E-22	0.77	Adipose - Subcutaneous
ENSG00000196735.7	*HLA-DQA1*	rs2647012	rs116393447	C>T	T/C	1.50E-10	-0.51	Adipose - Subcutaneous
ENSG00000196126.6	*HLA-DRB1*	rs2647012	rs116393447	C>T	T/C	1.40E-06	-0.38	Adipose - Subcutaneous
ENSG00000204338.4	*CYP21A1P*	rs2647012	rs116393447	C>T	T/C	4.10E-06	0.37	Adipose - Subcutaneous
ENSG00000179344.12	*HLA-DQB1*	rs2647012	rs116393447	C>T	T/C	5.60E-16	-0.86	Adipose - Visceral (Omentum)
ENSG00000229391.3	*HLA-DRB6*	rs2647012	rs116393447	C>T	T/C	9.00E-15	0.81	Adipose - Visceral (Omentum)
ENSG00000237541.3	*HLA-DQA2*	rs2647012	rs116393447	C>T	T/C	1.30E-12	0.76	Adipose - Visceral (Omentum)
ENSG00000223534.1	*HLA-DQB1-AS1*	rs2647012	rs116393447	C>T	T/C	7.20E-11	-0.7	Adipose - Visceral (Omentum)
ENSG00000179344.12	*HLA-DQB1*	rs2647012	rs116393447	C>T	T/C	3.00E-12	-0.88	Adrenal Gland
ENSG00000229391.3	*HLA-DRB6*	rs2647012	rs116393447	C>T	T/C	3.40E-10	0.82	Adrenal Gland
ENSG00000223534.1	*HLA-DQB1-AS1*	rs2647012	rs116393447	C>T	T/C	4.30E-09	-0.75	Adrenal Gland
ENSG00000248290.1	*TNXA*	rs2647012	rs116393447	C>T	T/C	4.90E-06	0.56	Adrenal Gland
ENSG00000237541.3	*HLA-DQA2*	rs2647012	rs116393447	C>T	T/C	6.90E-06	0.62	Adrenal Gland
ENSG00000179344.12	*HLA-DQB1*	rs2647012	rs116393447	C>T	T/C	3.20E-22	-0.85	Artery - Aorta
ENSG00000229391.3	*HLA-DRB6*	rs2647012	rs116393447	C>T	T/C	7.00E-15	0.74	Artery - Aorta
ENSG00000223534.1	*HLA-DQB1-AS1*	rs2647012	rs116393447	C>T	T/C	1.00E-13	-0.7	Artery - Aorta
ENSG00000196126.6	*HLA-DRB1*	rs2647012	rs116393447	C>T	T/C	1.30E-08	-0.48	Artery - Aorta
ENSG00000237541.3	*HLA-DQA2*	rs2647012	rs116393447	C>T	T/C	2.50E-08	0.48	Artery - Aorta
ENSG00000196735.7	*HLA-DQA1*	rs2647012	rs116393447	C>T	T/C	1.40E-07	-0.46	Artery - Aorta
ENSG00000204338.4	*CYP21A1P*	rs2647012	rs116393447	C>T	T/C	4.90E-06	0.45	Artery - Aorta
ENSG00000179344.12	*HLA-DQB1*	rs2647012	rs116393447	C>T	T/C	5.00E-14	-0.96	Artery - Coronary
ENSG00000223534.1	*HLA-DQB1-AS1*	rs2647012	rs116393447	C>T	T/C	4.70E-10	-0.83	Artery - Coronary
ENSG00000229391.3	*HLA-DRB6*	rs2647012	rs116393447	C>T	T/C	2.00E-08	0.73	Artery - Coronary
ENSG00000237541.3	*HLA-DQA2*	rs2647012	rs116393447	C>T	T/C	3.70E-07	0.59	Artery - Coronary
ENSG00000179344.12	*HLA-DQB1*	rs2647012	rs116393447	C>T	T/C	2.80E-41	-1	Artery - Tibial
ENSG00000223534.1	*HLA-DQB1-AS1*	rs2647012	rs116393447	C>T	T/C	5.10E-24	-0.78	Artery - Tibial
ENSG00000229391.3	*HLA-DRB6*	rs2647012	rs116393447	C>T	T/C	4.70E-22	0.75	Artery - Tibial
ENSG00000237541.3	*HLA-DQA2*	rs2647012	rs116393447	C>T	T/C	3.70E-20	0.66	Artery - Tibial
ENSG00000196735.7	*HLA-DQA1*	rs2647012	rs116393447	C>T	T/C	6.20E-16	-0.6	Artery - Tibial
ENSG00000196126.6	*HLA-DRB1*	rs2647012	rs116393447	C>T	T/C	7.50E-09	-0.38	Artery - Tibial
ENSG00000204338.4	*CYP21A1P*	rs2647012	rs116393447	C>T	T/C	5.30E-06	0.35	Artery - Tibial
ENSG00000179344.12	*HLA-DQB1*	rs2647012	rs116393447	C>T	T/C	9.30E-06	-0.84	Brain - Anterior cingulate cortex (BA24)
ENSG00000179344.12	*HLA-DQB1*	rs2647012	rs116393447	C>T	T/C	4.40E-13	-0.88	Brain - Caudate (basal ganglia)
ENSG00000223534.1	*HLA-DQB1-AS1*	rs2647012	rs116393447	C>T	T/C	1.50E-10	-0.77	Brain - Caudate (basal ganglia)
ENSG00000229391.3	*HLA-DRB6*	rs2647012	rs116393447	C>T	T/C	1.40E-06	0.65	Brain - Caudate (basal ganglia)
ENSG00000196735.7	*HLA-DQA1*	rs2647012	rs116393447	C>T	T/C	1.40E-06	-0.57	Brain - Caudate (basal ganglia)
ENSG00000196126.6	*HLA-DRB1*	rs2647012	rs116393447	C>T	T/C	1.70E-05	-0.48	Brain - Caudate (basal ganglia)
ENSG00000179344.12	*HLA-DQB1*	rs2647012	rs116393447	C>T	T/C	7.90E-12	-0.94	Brain - Cerebellar Hemisphere
ENSG00000179344.12	*HLA-DQB1*	rs2647012	rs116393447	C>T	T/C	3.60E-08	-0.66	Brain - Cerebellum
ENSG00000229391.3	*HLA-DRB6*	rs2647012	rs116393447	C>T	T/C	9.60E-07	0.62	Brain - Cerebellum
ENSG00000223534.1	*HLA-DQB1-AS1*	rs2647012	rs116393447	C>T	T/C	4.00E-06	-0.6	Brain - Cerebellum
ENSG00000204338.4	*CYP21A1P*	rs2647012	rs116393447	C>T	T/C	9.10E-06	0.6	Brain - Cerebellum
ENSG00000179344.12	*HLA-DQB1*	rs2647012	rs116393447	C>T	T/C	6.70E-09	-0.77	Brain - Cortex
ENSG00000223534.1	*HLA-DQB1-AS1*	rs2647012	rs116393447	C>T	T/C	8.30E-08	-0.76	Brain - Cortex
ENSG00000196735.7	*HLA-DQA1*	rs2647012	rs116393447	C>T	T/C	2.20E-07	-0.65	Brain - Cortex
ENSG00000237541.3	*HLA-DQA2*	rs2647012	rs116393447	C>T	T/C	2.10E-06	0.61	Brain - Cortex
ENSG00000179344.12	*HLA-DQB1*	rs2647012	rs116393447	C>T	T/C	1.30E-08	-0.76	Brain - Frontal Cortex (BA9)
ENSG00000223534.1	*HLA-DQB1-AS1*	rs2647012	rs116393447	C>T	T/C	5.80E-07	-0.7	Brain - Frontal Cortex (BA9)
ENSG00000237541.3	*HLA-DQA2*	rs2647012	rs116393447	C>T	T/C	1.70E-06	0.65	Brain - Frontal Cortex (BA9)
ENSG00000179344.12	*HLA-DQB1*	rs2647012	rs116393447	C>T	T/C	1.40E-10	-0.88	Brain - Hippocampus
ENSG00000196735.7	*HLA-DQA1*	rs2647012	rs116393447	C>T	T/C	7.30E-07	-0.6	Brain - Hippocampus
ENSG00000229391.3	*HLA-DRB6*	rs2647012	rs116393447	C>T	T/C	3.70E-06	0.68	Brain - Hippocampus
ENSG00000237541.3	*HLA-DQA2*	rs2647012	rs116393447	C>T	T/C	3.90E-06	0.48	Brain - Hippocampus
ENSG00000223534.1	*HLA-DQB1-AS1*	rs2647012	rs116393447	C>T	T/C	4.70E-06	-0.65	Brain - Hippocampus
ENSG00000179344.12	*HLA-DQB1*	rs2647012	rs116393447	C>T	T/C	1.70E-10	-0.91	Brain - Hypothalamus
ENSG00000229391.3	*HLA-DRB6*	rs2647012	rs116393447	C>T	T/C	3.00E-06	0.67	Brain - Hypothalamus
ENSG00000223534.1	*HLA-DQB1-AS1*	rs2647012	rs116393447	C>T	T/C	4.50E-06	-0.75	Brain - Hypothalamus
ENSG00000237541.3	*HLA-DQA2*	rs2647012	rs116393447	C>T	T/C	4.60E-06	0.68	Brain - Hypothalamus
ENSG00000179344.12	*HLA-DQB1*	rs2647012	rs116393447	C>T	T/C	2.70E-15	-1	Brain - Nucleus accumbens (basal ganglia)
ENSG00000223534.1	*HLA-DQB1-AS1*	rs2647012	rs116393447	C>T	T/C	7.20E-08	-0.7	Brain - Nucleus accumbens (basal ganglia)
ENSG00000237541.3	*HLA-DQA2*	rs2647012	rs116393447	C>T	T/C	1.60E-07	0.65	Brain - Nucleus accumbens (basal ganglia)
ENSG00000179344.12	*HLA-DQB1*	rs2647012	rs116393447	C>T	T/C	5.10E-10	-0.87	Brain - Putamen (basal ganglia)
ENSG00000223534.1	*HLA-DQB1-AS1*	rs2647012	rs116393447	C>T	T/C	2.40E-05	-0.65	Brain - Putamen (basal ganglia)
ENSG00000179344.12	*HLA-DQB1*	rs2647012	rs116393447	C>T	T/C	5.50E-20	-0.85	Breast - Mammary Tissue
ENSG00000229391.3	*HLA-DRB6*	rs2647012	rs116393447	C>T	T/C	5.30E-16	0.8	Breast - Mammary Tissue
ENSG00000223534.1	*HLA-DQB1-AS1*	rs2647012	rs116393447	C>T	T/C	4.60E-15	-0.72	Breast - Mammary Tissue
ENSG00000237541.3	*HLA-DQA2*	rs2647012	rs116393447	C>T	T/C	5.20E-12	0.68	Breast - Mammary Tissue
ENSG00000196735.7	*HLA-DQA1*	rs2647012	rs116393447	C>T	T/C	2.40E-06	-0.47	Breast - Mammary Tissue
ENSG00000204338.4	*CYP21A1P*	rs2647012	rs116393447	C>T	T/C	3.00E-05	0.32	Breast - Mammary Tissue
ENSG00000196126.6	*HLA-DRB1*	rs2647012	rs116393447	C>T	T/C	5.60E-06	-0.41	Cells - Transformed fibroblasts
ENSG00000179344.12	*HLA-DQB1*	rs2647012	rs116393447	C>T	T/C	2.00E-05	-0.4	Cells - Transformed fibroblasts
ENSG00000179344.12	*HLA-DQB1*	rs2647012	rs116393447	C>T	T/C	1.50E-16	-0.97	Colon - Sigmoid
ENSG00000223534.1	*HLA-DQB1-AS1*	rs2647012	rs116393447	C>T	T/C	1.90E-12	-0.84	Colon - Sigmoid
ENSG00000229391.3	*HLA-DRB6*	rs2647012	rs116393447	C>T	T/C	6.90E-08	0.67	Colon - Sigmoid
ENSG00000196735.7	*HLA-DQA1*	rs2647012	rs116393447	C>T	T/C	7.20E-07	-0.62	Colon - Sigmoid
ENSG00000237541.3	*HLA-DQA2*	rs2647012	rs116393447	C>T	T/C	4.90E-06	0.59	Colon - Sigmoid
ENSG00000179344.12	*HLA-DQB1*	rs2647012	rs116393447	C>T	T/C	6.50E-18	-0.94	Colon - Transverse
ENSG00000223534.1	*HLA-DQB1-AS1*	rs2647012	rs116393447	C>T	T/C	1.30E-15	-0.87	Colon - Transverse
ENSG00000237541.3	*HLA-DQA2*	rs2647012	rs116393447	C>T	T/C	1.50E-10	0.62	Colon - Transverse
ENSG00000229391.3	*HLA-DRB6*	rs2647012	rs116393447	C>T	T/C	1.80E-10	0.66	Colon - Transverse
ENSG00000196735.7	*HLA-DQA1*	rs2647012	rs116393447	C>T	T/C	1.40E-07	-0.52	Colon - Transverse
ENSG00000196126.6	*HLA-DRB1*	rs2647012	rs116393447	C>T	T/C	7.50E-07	-0.48	Colon - Transverse
ENSG00000229391.3	*HLA-DRB6*	rs2647012	rs116393447	C>T	T/C	2.20E-14	0.9	Esophagus - Gastroesophageal Junction
ENSG00000179344.12	*HLA-DQB1*	rs2647012	rs116393447	C>T	T/C	1.20E-09	-0.74	Esophagus - Gastroesophageal Junction
ENSG00000237541.3	*HLA-DQA2*	rs2647012	rs116393447	C>T	T/C	3.90E-09	0.73	Esophagus - Gastroesophageal Junction
ENSG00000223534.1	*HLA-DQB1-AS1*	rs2647012	rs116393447	C>T	T/C	1.40E-07	-0.57	Esophagus - Gastroesophageal Junction
ENSG00000179344.12	*HLA-DQB1*	rs2647012	rs116393447	C>T	T/C	6.20E-25	-0.92	Esophagus - Mucosa
ENSG00000229391.3	*HLA-DRB6*	rs2647012	rs116393447	C>T	T/C	2.80E-16	0.75	Esophagus - Mucosa
ENSG00000223534.1	*HLA-DQB1-AS1*	rs2647012	rs116393447	C>T	T/C	1.50E-14	-0.69	Esophagus - Mucosa
ENSG00000196735.7	*HLA-DQA1*	rs2647012	rs116393447	C>T	T/C	1.30E-11	-0.61	Esophagus - Mucosa

ENSG00000237541.3	*HLA-DQA2*	rs2647012	rs116393447	C>T	T/C	6.70E-10	0.5	Esophagus - Mucosa
ENSG00000196126.6	*HLA-DRB1*	rs2647012	rs116393447	C>T	T/C	2.20E-06	-0.41	Esophagus - Mucosa
ENSG00000179344.12	*HLA-DQB1*	rs2647012	rs116393447	C>T	T/C	5.80E-22	-0.91	Esophagus - Muscularis
ENSG00000229391.3	*HLA-DRB6*	rs2647012	rs116393447	C>T	T/C	2.60E-17	0.81	Esophagus - Muscularis
ENSG00000223534.1	*HLA-DQB1-AS1*	rs2647012	rs116393447	C>T	T/C	1.60E-15	-0.74	Esophagus - Muscularis
ENSG00000237541.3	*HLA-DQA2*	rs2647012	rs116393447	C>T	T/C	2.00E-11	0.66	Esophagus - Muscularis
ENSG00000204338.4	*CYP21A1P*	rs2647012	rs116393447	C>T	T/C	2.40E-06	0.37	Esophagus - Muscularis
ENSG00000196735.7	*HLA-DQA1*	rs2647012	rs116393447	C>T	T/C	1.80E-05	-0.43	Esophagus - Muscularis
ENSG00000244731.3	*C4A*	rs2647012	rs116393447	C>T	T/C	8.30E-05	0.24	Esophagus - Muscularis
ENSG00000179344.12	*HLA-DQB1*	rs2647012	rs116393447	C>T	T/C	1.60E-19	-0.95	Heart - Atrial Appendage
ENSG00000223534.1	*HLA-DQB1-AS1*	rs2647012	rs116393447	C>T	T/C	7.60E-15	-0.82	Heart - Atrial Appendage
ENSG00000229391.3	*HLA-DRB6*	rs2647012	rs116393447	C>T	T/C	2.90E-09	0.67	Heart - Atrial Appendage
ENSG00000237541.3	*HLA-DQA2*	rs2647012	rs116393447	C>T	T/C	8.60E-09	0.59	Heart - Atrial Appendage
ENSG00000196735.7	*HLA-DQA1*	rs2647012	rs116393447	C>T	T/C	9.00E-07	-0.53	Heart - Atrial Appendage
ENSG00000196126.6	*HLA-DRB1*	rs2647012	rs116393447	C>T	T/C	2.90E-05	-0.46	Heart - Atrial Appendage
ENSG00000179344.12	*HLA-DQB1*	rs2647012	rs116393447	C>T	T/C	6.20E-19	-0.95	Heart - Left Ventricle
ENSG00000229391.3	*HLA-DRB6*	rs2647012	rs116393447	C>T	T/C	1.80E-16	0.84	Heart - Left Ventricle
ENSG00000237541.3	*HLA-DQA2*	rs2647012	rs116393447	C>T	T/C	6.70E-08	0.53	Heart - Left Ventricle
ENSG00000223534.1	*HLA-DQB1-AS1*	rs2647012	rs116393447	C>T	T/C	9.30E-08	-0.56	Heart - Left Ventricle
ENSG00000179344.12	*HLA-DQB1*	rs2647012	rs116393447	C>T	T/C	1.00E-06	-0.67	Liver
ENSG00000229391.3	*HLA-DRB6*	rs2647012	rs116393447	C>T	T/C	2.80E-06	0.67	Liver
ENSG00000179344.12	*HLA-DQB1*	rs2647012	rs116393447	C>T	T/C	1.20E-35	-1	Lung
ENSG00000223534.1	*HLA-DQB1-AS1*	rs2647012	rs116393447	C>T	T/C	3.20E-23	-0.8	Lung
ENSG00000229391.3	*HLA-DRB6*	rs2647012	rs116393447	C>T	T/C	1.20E-20	0.72	Lung
ENSG00000237541.3	*HLA-DQA2*	rs2647012	rs116393447	C>T	T/C	4.50E-17	0.63	Lung
ENSG00000196735.7	*HLA-DQA1*	rs2647012	rs116393447	C>T	T/C	3.30E-13	-0.61	Lung
ENSG00000196126.6	*HLA-DRB1*	rs2647012	rs116393447	C>T	T/C	6.40E-08	-0.45	Lung
ENSG00000204338.4	*CYP21A1P*	rs2647012	rs116393447	C>T	T/C	2.70E-06	0.37	Lung
ENSG00000179344.12	*HLA-DQB1*	rs2647012	rs116393447	C>T	T/C	4.30E-30	-0.82	Muscle - Skeletal
ENSG00000229391.3	*HLA-DRB6*	rs2647012	rs116393447	C>T	T/C	3.00E-26	0.77	Muscle - Skeletal
ENSG00000237541.3	*HLA-DQA2*	rs2647012	rs116393447	C>T	T/C	1.80E-25	0.74	Muscle - Skeletal
ENSG00000223534.1	*HLA-DQB1-AS1*	rs2647012	rs116393447	C>T	T/C	2.30E-15	-0.61	Muscle - Skeletal
ENSG00000196735.7	*HLA-DQA1*	rs2647012	rs116393447	C>T	T/C	6.70E-07	-0.38	Muscle - Skeletal
ENSG00000196126.6	*HLA-DRB1*	rs2647012	rs116393447	C>T	T/C	6.30E-06	-0.32	Muscle - Skeletal
ENSG00000204287.9	*HLA-DRA*	rs2647012	rs116393447	C>T	T/C	5.80E-05	0.19	Muscle - Skeletal
ENSG00000179344.12	*HLA-DQB1*	rs2647012	rs116393447	C>T	T/C	5.30E-31	-0.97	Nerve - Tibial
ENSG00000229391.3	*HLA-DRB6*	rs2647012	rs116393447	C>T	T/C	2.50E-22	0.82	Nerve - Tibial
ENSG00000223534.1	*HLA-DQB1-AS1*	rs2647012	rs116393447	C>T	T/C	4.00E-20	-0.77	Nerve - Tibial
ENSG00000237541.3	*HLA-DQA2*	rs2647012	rs116393447	C>T	T/C	2.70E-17	0.68	Nerve - Tibial
ENSG00000196735.7	*HLA-DQA1*	rs2647012	rs116393447	C>T	T/C	2.80E-10	-0.54	Nerve - Tibial
ENSG00000196126.6	*HLA-DRB1*	rs2647012	rs116393447	C>T	T/C	1.90E-07	-0.41	Nerve - Tibial
ENSG00000204338.4	*CYP21A1P*	rs2647012	rs116393447	C>T	T/C	5.50E-06	0.38	Nerve - Tibial
ENSG00000179344.12	*HLA-DQB1*	rs2647012	rs116393447	C>T	T/C	3.20E-07	-0.9	Ovary
ENSG00000223534.1	*HLA-DQB1-AS1*	rs2647012	rs116393447	C>T	T/C	1.40E-05	-0.77	Ovary
ENSG00000179344.12	*HLA-DQB1*	rs2647012	rs116393447	C>T	T/C	1.20E-11	-0.79	Pancreas
ENSG00000229391.3	*HLA-DRB6*	rs2647012	rs116393447	C>T	T/C	8.20E-10	0.71	Pancreas
ENSG00000223534.1	*HLA-DQB1-AS1*	rs2647012	rs116393447	C>T	T/C	1.30E-07	-0.63	Pancreas
ENSG00000237541.3	*HLA-DQA2*	rs2647012	rs116393447	C>T	T/C	1.10E-05	0.48	Pancreas
ENSG00000229391.3	*HLA-DRB6*	rs2647012	rs116393447	C>T	T/C	5.60E-09	0.89	Pituitary
ENSG00000223534.1	*HLA-DQB1-AS1*	rs2647012	rs116393447	C>T	T/C	1.00E-08	-0.81	Pituitary
ENSG00000179344.12	*HLA-DQB1*	rs2647012	rs116393447	C>T	T/C	2.10E-08	-0.78	Pituitary
ENSG00000237541.3	*HLA-DQA2*	rs2647012	rs116393447	C>T	T/C	1.70E-06	0.66	Pituitary
ENSG00000179344.12	*HLA-DQB1*	rs2647012	rs116393447	C>T	T/C	9.60E-13	-0.95	Prostate
ENSG00000237541.3	*HLA-DQA2*	rs2647012	rs116393447	C>T	T/C	1.90E-09	0.75	Prostate
ENSG00000229391.3	*HLA-DRB6*	rs2647012	rs116393447	C>T	T/C	9.50E-09	0.81	Prostate
ENSG00000223534.1	*HLA-DQB1-AS1*	rs2647012	rs116393447	C>T	T/C	5.20E-08	-0.72	Prostate
ENSG00000179344.12	*HLA-DQB1*	rs2647012	rs116393447	C>T	T/C	3.90E-22	-0.91	Skin - Not Sun Exposed (Suprapubic)
ENSG00000223534.1	*HLA-DQB1-AS1*	rs2647012	rs116393447	C>T	T/C	5.00E-13	-0.7	Skin - Not Sun Exposed (Suprapubic)
ENSG00000229391.3	*HLA-DRB6*	rs2647012	rs116393447	C>T	T/C	5.90E-13	0.72	Skin - Not Sun Exposed (Suprapubic)
ENSG00000196735.7	*HLA-DQA1*	rs2647012	rs116393447	C>T	T/C	7.60E-09	-0.55	Skin - Not Sun Exposed (Suprapubic)
ENSG00000196126.6	*HLA-DRB1*	rs2647012	rs116393447	C>T	T/C	1.10E-05	-0.43	Skin - Not Sun Exposed (Suprapubic)
ENSG00000237541.3	*HLA-DQA2*	rs2647012	rs116393447	C>T	T/C	2.60E-05	0.34	Skin - Not Sun Exposed (Suprapubic)
ENSG00000179344.12	*HLA-DQB1*	rs2647012	rs116393447	C>T	T/C	1.40E-35	-0.96	Skin - Sun Exposed (Lower leg)
ENSG00000223534.1	*HLA-DQB1-AS1*	rs2647012	rs116393447	C>T	T/C	2.00E-24	-0.79	Skin - Sun Exposed (Lower leg)
ENSG00000229391.3	*HLA-DRB6*	rs2647012	rs116393447	C>T	T/C	4.60E-22	0.76	Skin - Sun Exposed (Lower leg)
ENSG00000196735.7	*HLA-DQA1*	rs2647012	rs116393447	C>T	T/C	1.20E-13	-0.6	Skin - Sun Exposed (Lower leg)
ENSG00000237541.3	*HLA-DQA2*	rs2647012	rs116393447	C>T	T/C	1.50E-10	0.39	Skin - Sun Exposed (Lower leg)
ENSG00000196126.6	*HLA-DRB1*	rs2647012	rs116393447	C>T	T/C	1.50E-07	-0.42	Skin - Sun Exposed (Lower leg)
ENSG00000204338.4	*CYP21A1P*	rs2647012	rs116393447	C>T	T/C	3.90E-06	0.35	Skin - Sun Exposed (Lower leg)
ENSG00000196301.3	*HLA-DRB9*	rs2647012	rs116393447	C>T	T/C	2.90E-05	0.36	Skin - Sun Exposed (Lower leg)
ENSG00000179344.12	*HLA-DQB1*	rs2647012	rs116393447	C>T	T/C	1.50E-08	-0.86	Spleen
ENSG00000229391.3	*HLA-DRB6*	rs2647012	rs116393447	C>T	T/C	1.50E-07	0.82	Spleen
ENSG00000223534.1	*HLA-DQB1-AS1*	rs2647012	rs116393447	C>T	T/C	3.00E-06	-0.76	Spleen
ENSG00000237541.3	*HLA-DQA2*	rs2647012	rs116393447	C>T	T/C	5.40E-06	0.64	Spleen
ENSG00000179344.12	*HLA-DQB1*	rs2647012	rs116393447	C>T	T/C	4.20E-15	-0.92	Stomach
ENSG00000223534.1	*HLA-DQB1-AS1*	rs2647012	rs116393447	C>T	T/C	3.90E-10	-0.75	Stomach
ENSG00000229391.3	*HLA-DRB6*	rs2647012	rs116393447	C>T	T/C	4.70E-09	0.61	Stomach
ENSG00000237541.3	*HLA-DQA2*	rs2647012	rs116393447	C>T	T/C	6.40E-08	0.54	Stomach
ENSG00000179344.12	*HLA-DQB1*	rs2647012	rs116393447	C>T	T/C	2.00E-15	-0.87	Testis
ENSG00000223534.1	*HLA-DQB1-AS1*	rs2647012	rs116393447	C>T	T/C	1.50E-10	-0.68	Testis
ENSG00000237541.3	*HLA-DQA2*	rs2647012	rs116393447	C>T	T/C	1.80E-10	0.69	Testis
ENSG00000229391.3	*HLA-DRB6*	rs2647012	rs116393447	C>T	T/C	2.10E-09	0.62	Testis
ENSG00000196735.7	*HLA-DQA1*	rs2647012	rs116393447	C>T	T/C	1.30E-07	-0.59	Testis
ENSG00000204338.4	*CYP21A1P*	rs2647012	rs116393447	C>T	T/C	1.80E-06	0.47	Testis
ENSG00000196126.6	*HLA-DRB1*	rs2647012	rs116393447	C>T	T/C	7.30E-05	-0.43	Testis
ENSG00000179344.12	*HLA-DQB1*	rs2647012	rs116393447	C>T	T/C	1.70E-29	-0.9	Thyroid
ENSG00000223534.1	*HLA-DQB1-AS1*	rs2647012	rs116393447	C>T	T/C	1.60E-21	-0.73	Thyroid
ENSG00000229391.3	*HLA-DRB6*	rs2647012	rs116393447	C>T	T/C	5.00E-21	0.75	Thyroid
ENSG00000237541.3	*HLA-DQA2*	rs2647012	rs116393447	C>T	T/C	6.60E-19	0.65	Thyroid
ENSG00000204338.4	*CYP21A1P*	rs2647012	rs116393447	C>T	T/C	5.30E-09	0.35	Thyroid
ENSG00000196735.7	*HLA-DQA1*	rs2647012	rs116393447	C>T	T/C	7.50E-09	-0.47	Thyroid
ENSG00000196126.6	*HLA-DRB1*	rs2647012	rs116393447	C>T	T/C	2.80E-06	-0.38	Thyroid
ENSG00000179344.12	*HLA-DQB1*	rs2647012	rs116393447	C>T	T/C	4.50E-09	-1.2	Uterus
ENSG00000223534.1	*HLA-DQB1-AS1*	rs2647012	rs116393447	C>T	T/C	3.50E-06	-0.92	Uterus
ENSG00000229391.3	*HLA-DRB6*	rs2647012	rs116393447	C>T	T/C	4.90E-06	0.8	Uterus
ENSG00000229391.3	*HLA-DRB6*	rs2647012	rs116393447	C>T	T/C	1.00E-07	0.87	Vagina
ENSG00000179344.12	*HLA-DQB1*	rs2647012	rs116393447	C>T	T/C	6.10E-07	-0.82	Vagina

Ref/Alt Reference and alternative allele of the corresponding SNP
Effect size calculated based on alternative allele of SNP

Supplementary Table S5. Comparison of NHL SNPs association across different studies

SNP	CHR	GRCh38.p2	Study	Population	Disease type	MAF Controls	Odds ratio
rs6457327 C>A	6	31106253	Skibola et al., 2009	Caucasian	Folicular lymphoma	0.38	OR_{FL}=0.59 (0.50–0.70)
			Conde et al., 2010	Caucasian	Folicular lymphoma	0.48	OR_{FL}=0.68 (0.58-0.79)
			Malaysian NHL	Malay	All Non-Hodgkin lymphoma	0.41	OR_{NHL}=0.95 (0.74-1.22)
			Malaysian NHL	Chinese	All Non-Hodgkin lymphoma	0.35	OR_{NHL}=0.86 (0.66-1.12)
rs9271100 C>T	6	32608701	Han et al., 2009	Chinese Han	Lupus erythematosus	NR	OR_{LUP}=1.9 (1.59-2.27)
			Liu et al., 2015	Chinese Han	Leprosy	0.22	OR_{LEP}=1.68
			Malaysian NHL	Malay	All Non-Hodgkin lymphoma	0.32	OR_{NHL}=1.07 (0.83-1.39)
			Malaysian NHL	Chinese	All Non-Hodgkin lymphoma	0.17	OR_{NHL}=1.28 (0.92-1.78)
rs2647012 C>T	6	32696681	Smedby et al., 2011	Caucasian	Folicular lymphoma	0.48	OR_{FL}=0.64 (0.58-0.70)
			Malaysian NHL	Malay	All Non-Hodgkin lymphoma	0.18	OR_{NHL}=0.54 (0.37-0.77)
			Malaysian NHL	Chinese	All Non-Hodgkin lymphoma	0.22	OR_{NHL}=0.96 (0.7-1.31)
rs10484561 T>G	6	32697643	Conde et al., 2010	Caucasian	Folicular lymphoma	0.11	OR_{FL}=1.95 (1.72-2.22)
			Malaysian NHL	Malay	All Non-Hodgkin lymphoma	0.14	OR_{NHL}=1.31 (0.94-1.82)
			Malaysian NHL	Chinese	All Non-Hodgkin lymphoma	0.03	OR_{NHL}=1.14 (0.56-2.31)

NR- No record
